# Effects of a wind farm installation on the understory bat community of a highly biodiverse tropical region in Mexico

**DOI:** 10.7717/peerj.3424

**Published:** 2017-06-15

**Authors:** Miguel Briones-Salas, Mario C. Lavariega, Claudia E. Moreno

**Affiliations:** 1 Centro Interdisciplinario de Investigación Para el Desarrollo Integral Regional, Unidad Oaxaca, Instituto Politécnico Nacional, Santa Cruz Xoxocotlán, Oaxaca, Mexico; 2 Centro de Investigaciones Biológicas, Instituto de Ciencias Básicas e Ingeniería, Universidad Autónoma del Estado de Hidalgo, Mineral de la Reforma, Hidalgo, Mexico

**Keywords:** Wind energy, Turbines, Resilience, Chiroptera, Phylogenetic diversity, Isthmus of Tehuantepec, Functional diversity, Species diversity, Species richness, Oaxaca

## Abstract

Wind energy has rapidly become an important alternative among renewable energies, and it is generally considered clean. However, little is known about its impact at the level of ecological communities, especially in biodiversity hotspots. The Isthmus of Tehuantepec is a highly biodiverse region in Mesoamerica, and has the highest potential for generating wind energy in Mexico. To assess the effects of installing a wind farm on the understory bat community in a landscape of fragmented habitat, we assessed its diversity and composition over four stages of installation (site preparation, construction, and two stages of operation). We captured 919 bats belonging to 22 species. Species richness, functional diversity and phylogenetic diversity decreased during construction and the first stage of operation. However, these components of biodiversity increased during the second stage of operation, and species composition began to resemble that of the site preparation stage. No species considered as sensitive to disturbance was recorded at any stage. This is the first study to reveal the diversity of a Neotropical bat community after wind turbines begin to operate.

## Introduction

To mitigate global climate change by reducing greenhouse gas emissions, energy generated from renewable resources has gained interest as an alternative to that produced from fossil sources (Pazheri, Othman & Malik, 2014). Wind energy is a renewable source considered safe and clean because it does not have a severe impact on the atmosphere and health, and in recent decades its use has increased considerably around the world (Resch et al., 2008; Moriarty & Honnery, 2012). Currently, more than 100 countries use wind energy (WWEA, 2015). In Latin America the production of wind energy is relatively recent, Brazil (5,962 megawatts of installed capacity) and Mexico (2,621 megawatts of installed capacity) stand out in Latin America (SENER, 2015; WWEA, 2015), and are also countries with high levels of biodiversity (Mittermeier et al., 1998).

While wind farms do not emit greenhouse gases, they negatively impact biological populations, mainly by killing flying animals (bats and birds) that collide with blades and towers; presenting barriers to migratory routes; modifying habitats during the construction of roads, towers, and platforms; altering the landscape; and making noise (Arnett et al., 2007; Baerwald & Barclay, 2009; Garvin et al., 2011). Most of the studies addressing the impact of wind farms on bats by collision have been carried out in Europe and USA at the population level (Arnett et al., 2007; Kunz et al., 2007; Rydell et al., 2010; Cryan et al., 2014; Arnett et al., 2016). Only recently have Ferri, Battisti & Soccini (2016) analyzed this impact at the community level for a Mediterranean mountain landscape in central Italy.

In the Neotropics there are few studies analyzing bat diversity on wind farms or the effects of turbines on these organisms. Recent studies in Brazil (Barros, Gastal de Magalhaes & Rui, 2015) and Puerto Rico (Rodríguez-Durán & Feliciano-Robles, 2015) describe mortality caused by collision with turbines after their construction. In Mexico studies have focused on assessing bat species richness near wind farms (Briones-Salas, Peralta-Pérez & García-Luis, 2013; Muñoz et al., 2016), documenting the main scavengers that remove bat carcasses (Villegas-Patraca et al., 2012), and analyzing the effect of the spatial attributes of vegetation cover near turbines on species composition and bat fatalities (Bolívar-Cimé et al., 2016).

The first commercial wind farm in Mexico began operating in 1994 on the Isthmus of Tehuantepec, in the state of Oaxaca, southern Mexico. By June 2015, there were 30 wind farms operating in Mexico, and it is expected that the country will produce 11,585 megawatts by 2028, 4.42 times its current production (SENER, 2015). Oaxaca has the greatest wind power capacity in the country. The Isthmus of Tehuantepec can produce a lot of wind energy because of the strong winds year round that peak from November to February; the latter are known as *nortes* or *tehuanos* and reach 8–12 m/s (Elliott et al., 2004). In this region there are 20 wind farms operating, which together are known as the Aeolian Corridor of the Isthmus of Tehuantepec (Castillo, 2011).

The Isthmus of Tehuantepec is an area of exceptionally high biodiversity, endemism, and population differentiation for several taxa (Peterson, Soberón & Sánchez-Cordero, 1999), and it lies within the Mesoamerican hotspot (Olson & Dinerstein, 2002). Of the 150 species of mammals on the Isthmus of Tehuantepec in Oaxaca, 72 of them are bats (Briones-Salas, Cortés-Marcial & Lavariega, 2015). Neotropical bats are a diverse group that plays important roles in ecological processes (e.g., insect predators, pollinators, and seed dispersers; Ghanem & Voigt, 2012. They offer an interesting group for studying habitat disturbance García-Morales, Badano & Moreno, 2013; Bolívar-Cimé et al., 2014; Ripperger et al., 2015).

To assess the effect of wind farm installation on the understory bat community of the Isthmus of Tehuantepec, we carried out a long-term study analyzing the biodiversity and composition of a fragmented landscape. We sampled bats over four stages of the installation: site preparation, construction of the wind farm, and two operational stages (immediate and delayed effects). Specifically, we assessed species richness, abundance, species diversity, functional diversity, phylogenetic diversity and species composition at each of the four stages. Some studies in the Neotropics have found that species diversity is not always a useful measure to assess changes in bat communities (Medina et al., 2007; Harvey & Villalobos, 2007). Thus, in addition to species diversity, we are including other dimensions of biodiversity, such as functional and phylogenetic diversity, in order to assess those changes. The assessment of functional and phylogenetic diversity has provided a more comprehensive assessment of environmental and spatial mechanisms that drive the assembly of bat communities in human-modified landscapes (Cisneros, Fagan & Willing, 2014).

Our focus was the bat community that flies in the understory and is not directly affected per se by turbine collisions, but rather by habitat changes related to the farm installation. Given that high biodiversity in ecological communities is positively related to ecosystem resilience after disturbance (Gunderson, 2000), and considering the high diversity of bats in the region, we do not expect the magnitude of the disturbance to have a significant impact on the bat community.

## Materials and Methods

### Study area

**Figure 1:**
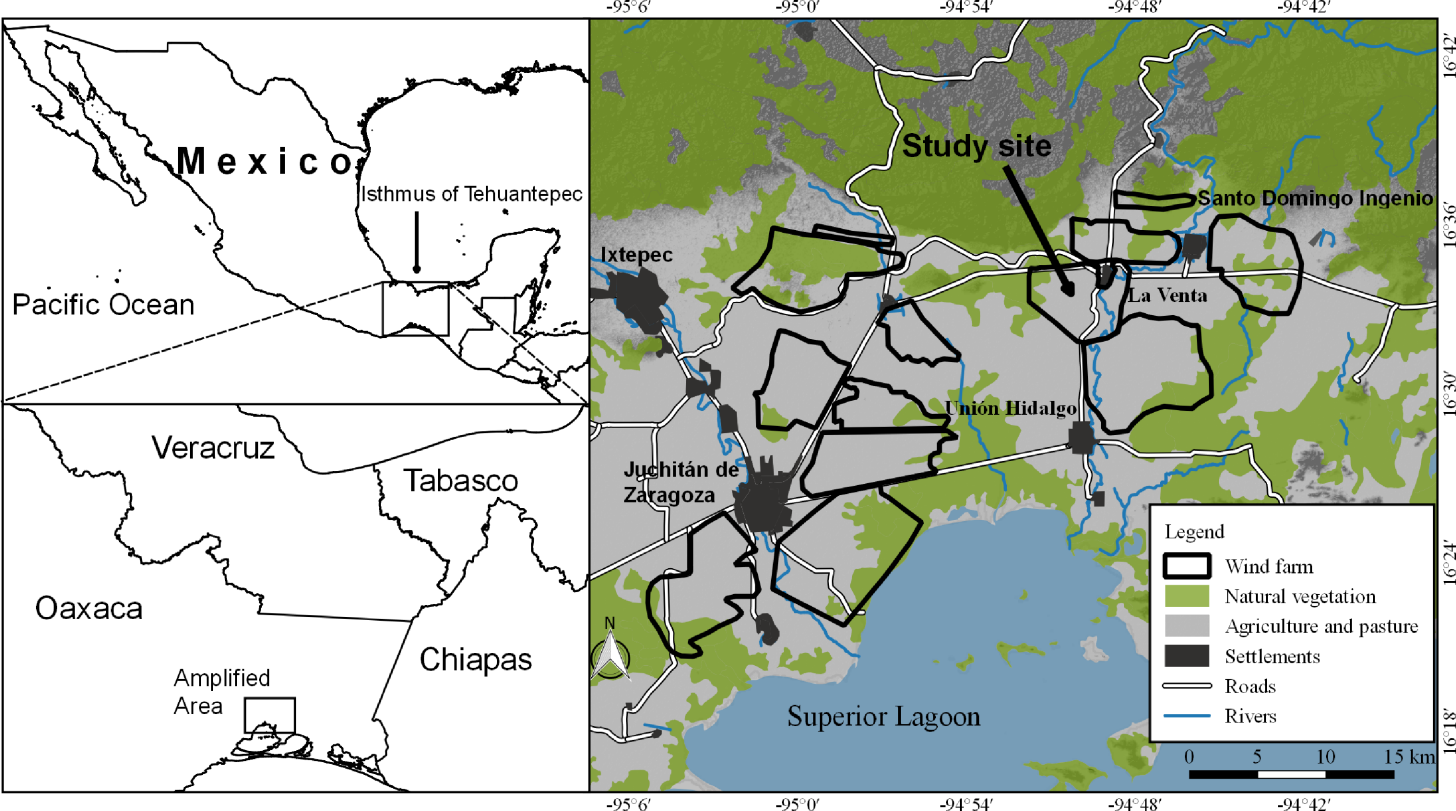
Study area. Location of sampling area on the Isthmus of Tehuantepec, Oaxaca, southern Mexico.

The study was done in La Venta, southeastern Oaxaca (16°31′–16°34′N, 94°48′–94°51′W; 25 m a.s.l.), in the municipality of Juchitán de Zaragoza (Fig. 1). The climate is warm sub-humid with a mean annual temperature of 22 °C and the temperature of the coldest month is 18 °C. During the driest month precipitation is 0–60 mm, and mean annual precipitation is 1,000 mm (Trejo, 2004). The natural vegetation is fragmented, with corn and sorghum fields and cattle pastures making up the landscape matrix, along with remnants of tropical deciduous and subdeciduous forest and secondary, halophilous and riparian vegetation along the Chicapa River (INEGI, 2013; Muñoz et al., 2016). There is also a network of irrigation channels.

We worked at a wind farm installed between 2007 and 2010. It has 167 wind turbines (SENER, 2015). Our study comprised four stages: (1) Site preparation, when the farm was delimited and the first roads constructed, characterized by low but continuous human activity; (2) The construction stage when the turbines, underground fiber optics network and a substation were installed. More roads and drainage along them were built, and mounting platforms were also installed. Human activity was intense; (3) and (4) Two operational stages, 15 and 30 months after construction, when the turbines were functioning, fences had been built and technicians frequently checked the physical conditions of each component in the turbines, in addition to doing preventative maintenance. Human activity decreased but was still regular (AMDEE, 2016).

### Estimation of habitat modification

To estimate habitat modification related to the wind farm installation, we assessed landscape composition and configuration in a 1 km buffer around the wind farm (47.30 ha). We made a non-supervised classification of 2008, 2013 and 2015 Landsat remote sensing images, corresponding at the beginning, middle and at the end of this study, with the ISODATA algorithm, using the QGis 2.6.1 software. We used 30 coverage classes and grouped them into forest cover (including forests, secondary vegetation and riparian corridors) and non-forest cover (agriculture, urban settlements, roads, etc.). Then, we estimated landscape structure parameters (forest cover area, number of patches and edge length).

### Sampling design

We carried out the study between 2007 and 2014 monitoring the bat community during the four stages described above. For each stage we sampled bats monthly (three or four nights per month) with 6–12 mist nets (12 m long) (see Tables S1 and S2, for details on netting effort). Bats were sampled from April 2007 to March 2008 during the site preparation stage. During the construction stage we sampled from November 2010 to August 2011. During operational stage 1 (immediate effect) we sampled 15 months after construction, from December 2012 to September 2013. During operational stage 2 (delayed effect) we sampled 30 months after construction, from March to December 2014. We sampled bats over 10 months each stage, and then pooled these data including both rainy and dry periods of the year at each stage. In this way, we account for the potential temporal variability in bat communities, and assume that seasonality did not influence our comparisons among stages.

We hung nets near water and trails among vegetation, 1.5–5 m above the ground. Thus, sampling was directed towards understory bats, especially families Phyllostomidae and Mormoopidae, that are easily caught with mist nets. Our study is not focused on bats directly impacted by turbine collisions. High flying aerial insectivores (Emballonuridae, Molossidae and Vespertilionidae families) are less likely to be caught in the understory and can be underrepresented by the sampling method (Galindo-González, 2004).

We placed nets in different locations during each sampling stage, to include heterogeneous areas within the landscape and when possible, trap locations remained the same over years. Nets were open for 7–8 h each night (see Tables S1 and S2, for details on bat sampling) and checked every 30 min. We did not sample at least three days before and after full moon, to avoid capture bias due to the bat lunar phobia (Saldaña-Vázquez & Munguía-Rosas, 2013). There were no extreme climatic events during the years of sampling: mean wind velocity per year ranged from 10 to 11 m/s; mean monthly temperature per year was 24–25 °C, and mean monthly relative humidity per year ranged from 78 to 80%.

We carried out bat sampling with permission of the Mexican Secretaría del Medio Ambiente y Recursos Naturales (FAUT-0037). We handled bats according to the guidelines of the American Society of Mammalogists for the use of wild mammals in research (Gannon & Sikes, 2007). We identified bats to species using taxonomic keys (Álvarez, Álvarez-Castañeda & López, 1994; Medellín, Arita & Sánchez, 2008), and updated species names according to Ramírez-Pulido et al. (2014). We deposited some voucher specimens in the Mammal Collection of CIIDIR-Oaxaca, IPN (OAX-MA 026.04.97), but the majority of the bats were released where captured.

### Data analysis

First, we assessed inventory completeness to determine if sampling effort at each stage was sufficient to capture an adequate representation of the species that could be captured with our sampling method. To this end, we calculated sample coverage (*Cm*), which measures sample completeness taking into account the total number of individuals captured and the number of rare species. The sampling deficit (1−*Cm*) represents the probability that the next individual captured will belong to a species that has not been recorded previously in the inventory (Chao & Jost, 2012), i.e., it estimates the proportion of the total individuals in the assemblage that belong to undetected species.

We compared total species richness among sampling stages using species accumulation curves. To standardize sampling effort we used the individual-based interpolation (rarefaction) and extrapolation multinomial model, with 95% unconditional confidence intervals (Colwell et al., 2012). This procedure uses a unified statistical framework to plot a unified species accumulation curve to the maximum number of individuals in the stage with the greatest bat abundance. We calculated the species accumulation curves, extrapolations and their SD in EstimateS 9.1 software (Colwell, 2013).

To compare bat biodiversity between construction stages we measured different components: species diversity, phylogenetic diversity and functional diversity. We calculated species diversity as the true diversity order 1 (exponential of Shannon’s entropy index), which measures the effective number of species taking into account all the species according to their relative abundance in the community (Jost, 2006).

We used the PD index to measure phylogenetic diversity (Faith, 1992; Faith, 2016), which measures the distance between pairs of species as the length of their branches in the phylogenetic tree of the group. A high PD value indicates a high degree of evolutionary diversification among species. For this, we used the phylogeny of mammals proposed by Bininda-Emonds et al. (2007). We also calculated phylogenetic variability with the PSV index of Helmus et al. (2007), to determine the degree of phylogenetic relatedness among the species caught. This index has a value of 1 at maximum evolutionary variability, and decreases toward zero as variability is lost and species are more related.

We measured functional diversity (FD) with the FD index (Petchey & Gaston, 2002; Petchey & Gaston, 2006), which is based on the phylogenetic diversity (PD) index of Faith (1992), but uses a functional dendrogram instead of a phylogeny. For this, we used four functional traits (Table 1), getting values from Bonaccorso (1979), Ceballos & Oliva (2005), and Jung & Kalko (2011a): size (mean forearm length, mm), weight (g), diet (frugivorous, hematophagous, nectarivorous or insectivorous), and flight height (high: more than 8 m above the ground, medium: 3–8 m, or low: below 3 m). We first transformed diet and flight height into dummy variables, and then we standardized all variables. We also calculated functional dispersion with the FDis index (Laliberté & Legendre, 2010). FDis measures the mean distance from each species to the centroid of all the species in a functional space, weighted by their relative abundances. FDis ranges from zero when there is only one species in the community, and increases as the distance of species from the centroid increases. We calculated all the phylogenetic and functional diversity indices in the FDiversity program (Casanoves et al., 2011).

**Table 1:** Number of bats captured over the four stages of wind farm installation in the Mexican tropics.

	Prep	Cons	Oper1	Oper2	Ind	Size (mm)	Weight (g)	Height		Diet	
Family Molossidae											
*Molossus rufus*	0	3	0	0	3	50	29	high		insectivorous	
*Molossus molossus*	0	0	1	3	4	40	17	high		insectivorous	
Family Mormoopidae											
*Mormoops megalophylla*	6	4	2	0	12	55	13	high		insectivorous	
*Pteronotus davyi*	4	3	0	0	7	45	7	high		insectivorous	
*Pteronotus parnellii*	66	9	0	2	77	58	19	low		insectivorous	
Family Phyllostomidae											
*Carollia subrufa*	2	0	0	0	2	39	14	medium		frugivorous	
*Desmodus rotundus*	5	0	0	0	5	61	31	low		hematophagous	
*Glossophaga commissarisi*	35	3	0	20	58	36	11	low		nectarivorous	
*Glossophaga leachii*	48	4	0	5	57	36	12	low		nectarivorous	
*Glossophaga morenoi*	29	4	8	13	54	35	11	low		nectarivorous	
*Glossophaga soricina*	37	4	10	4	55	36	11	low		nectarivorous	
*Leptonycteris yerbabuenae*	5	6	4	2	17	54	25	low		nectarivorous	
*Artibeus jamaicensis*	87	12	34	116	249	61	48	high		frugivorous	
*Artibeus lituratus*	38	7	68	50	163	66	57	high		frugivorous	
*Dermanura phaeotis*	1	0	0	0	1	37	11	high		frugivorous	
*Centurio senex*	0	1	0	2	3	44	17	medium		frugivorous	
*Uroderma bilobatum*	3	4	4	4	15	42	18	low		frugivorous	
*Sturnira hondurensis*	43	2	11	12	68	40	19	low		frugivorous	
*Sturnira parvidens*	11	13	10	14	48	40	18	low		frugivorous	
Family Vespertilionidae											
*Lasiurus blossevillii*	1	0	0	0	1	40	8	medium		insectivorous	
*Lasiurus intermedius*	1	0	0	1	2	45	19	medium		insectivorous	
*Rhogeessa parvula*	3	10	2	3	18	33	5	low		insectivorous	
Total number of individuals	425	89	154	251	919						
Total number of species	19	16	11	15	22						

**Notes.**

Prep preparation Cons construction Oper1 first stage of operation (immediate effect) Oper2 second stage of operation (delayed effect) Ind total number of individuals captured

Size (mean forearm length), Weight, Height (flight height) and Diet are functional traits whose values were obtained from literature (Bonaccorso, 1979; Ceballos & Oliva, 2005; Jung & Kalko, 2011a).

Finally, we used Principal Coordinates Analysis (PCoA, PCORD, McCune & Mefford, 2011) to compare species composition and the possible species turnover among the four sampling stages, in a space defined by their distance relationships. As a distance measure we used the Bray–Curtis dissimilarity index to include both species and their abundances.

## Results

Total forest cover area decreased from 12 km^2^ in 2008 to 10 km^2^ in 2013, and finally 9 km^2^ in 2015, this represent the 26, 21 and 18% of landscape, respectively. The number of forest patches remained almost the same (403 in 2008, 408 in 2013, and 415 in 2015) and the edge length decreased from 349 to 335 and 298 km.

We captured 919 bats comprising 22 species and four families: Molossidae (two species), Mormoopidae (three species), Phyllostomidae (14 species) and Vespertilionidae (three species). There were eight insectivore species, eight frugivores, five nectarivores and one hematophague. *Leptonycteris yerbabuenae* is the only species considered vulnerable (IUCN, 2015), and *Glossophaga morenoi* is a Mexican endemic species (SEMARNAT, 2010; Table 1). During all stages, the number of species captured was 99% of that predicted by the sample coverage estimate (*Cm*). Based on sampling efficiency, the data appeared to be a reliable sample of bat diversity at each stage.

**Figure 2:**
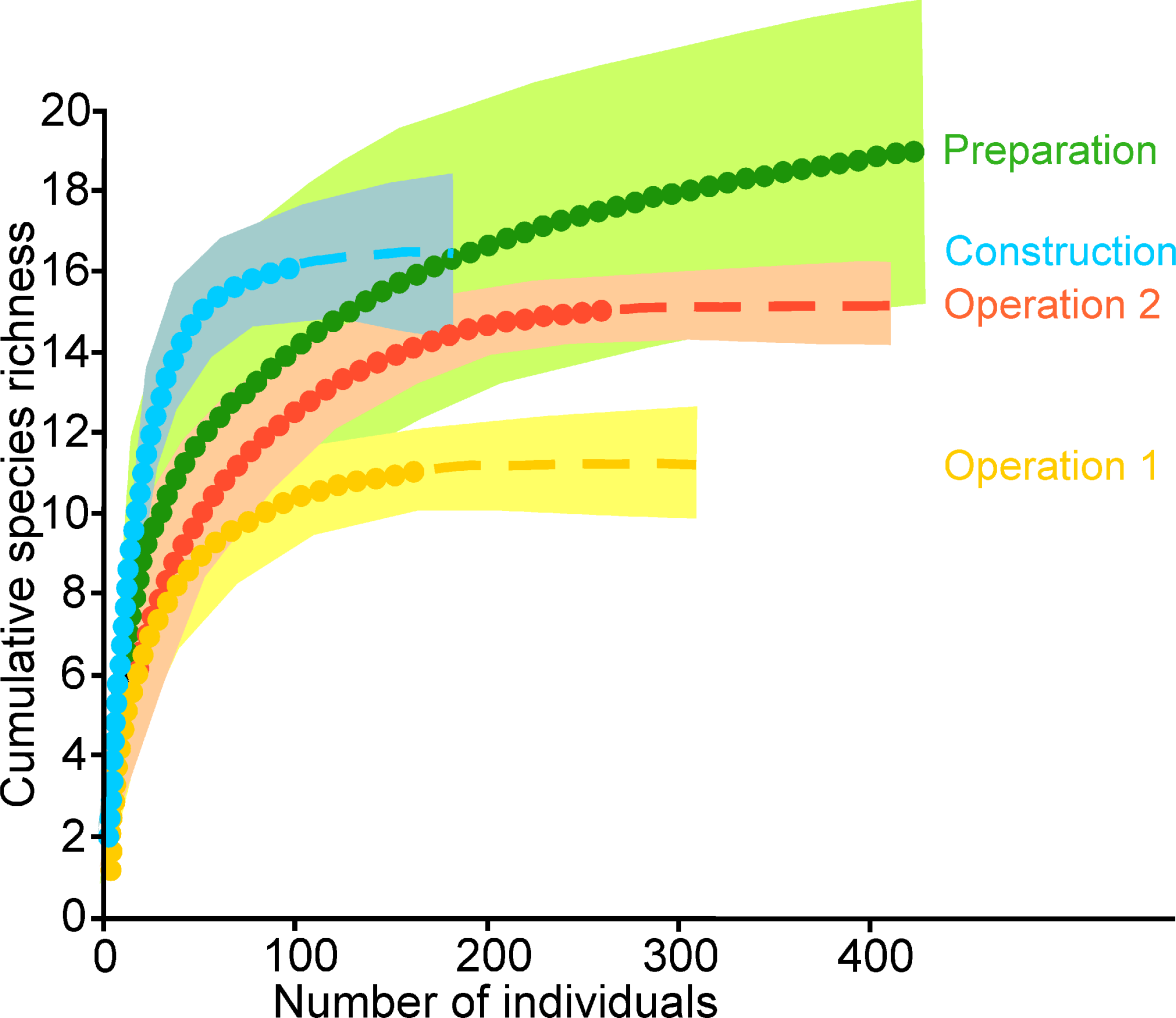
Bat species accumulation curves. Accumulation curves are drawn for four sampling stages during the installation of a wind farm on the Isthmus of Tehuantepec, southern Mexico. Circles are the recorded richness curves, and discontinuous lines are extrapolation models for increasing numbers of individuals. Shaded areas indicate 95% confidence intervals.

**Figure 3:**
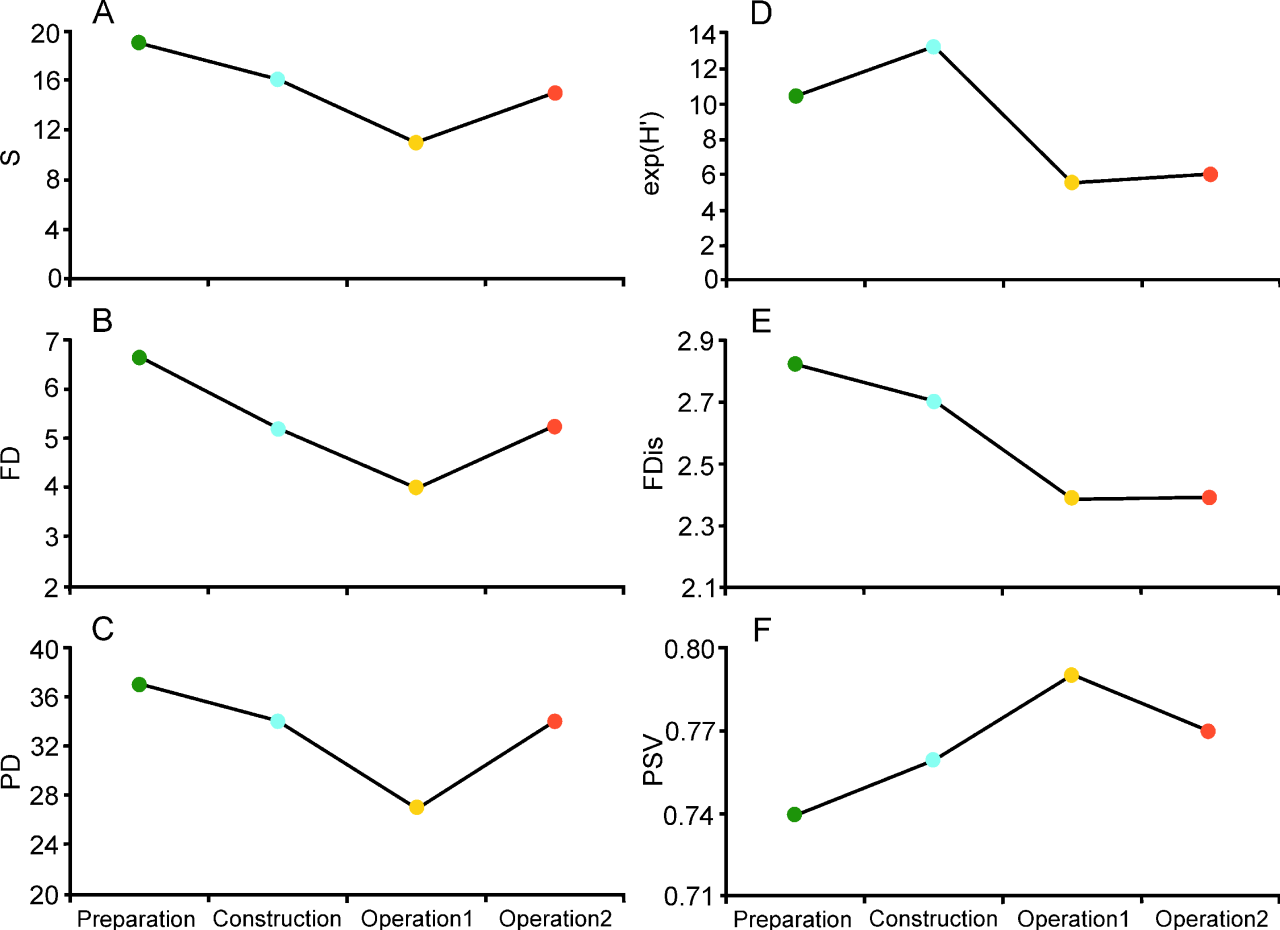
Bat diversity for four sampling stages during a wind farm installation on the Isthmus of Tehuantepec. Bat parameters calculated are species richness (S), functional diversity (FD), phylogenetic diversity (PD), species diversity (exponential of Shannon index), functional dispersion (FDis) and phylogenetic variability (PSV). See the Methods section for references.

We recorded more individuals and species in the preparation stage (Table 1 and Fig. 2). During the construction stage the capture rate decreased notably (Table 1), while species richness remained constant (Fig. 3A). During operational stage 1, richness was significantly lower than that of the other stages (Fig. 3A) and in operational stage 2 both the number of individuals and species increased (Table 1; Fig. 2), though richness was still lower than during preparation (Fig. 3A).

**Figure 4:**
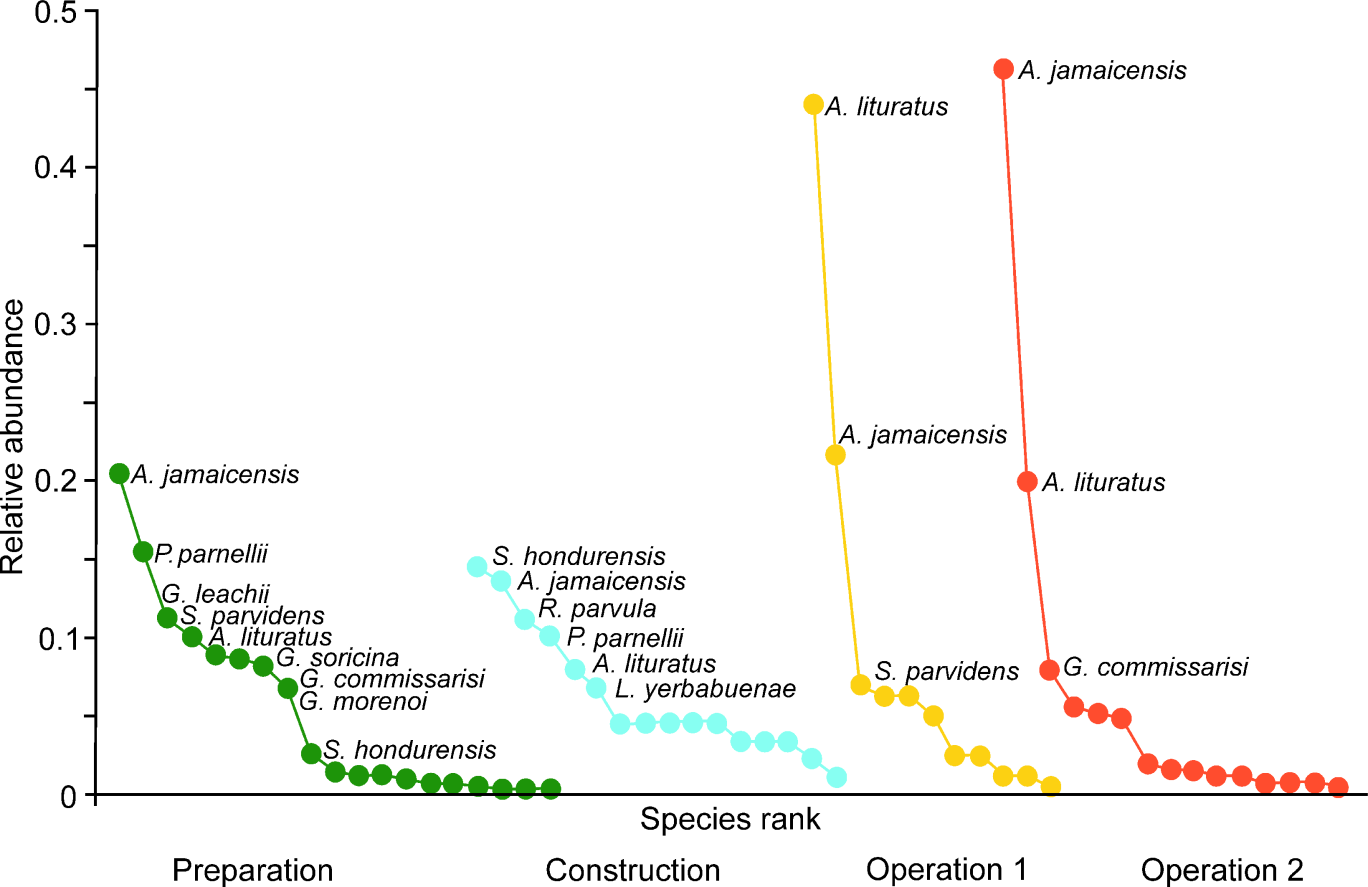
Rank dominance plots for bat species captured over four sampling stages during a wind farm installation on the Isthmus of Tehuantepec. For each stage, species are ranked by the number of individuals captured. Only the most abundant species are labeled.

Both functional diversity (FD) and phylogenetic diversity (PD) followed the same trend as species richness over the four stages (Figs. 3A–3C). However, species diversity increased during construction, and was low during the two operational stages (Fig. 3D). Similarly, functional dispersion (FDis) was low during the operational stages (Fig. 3E). In contrast, phylogenetic variability was highest during operational stage 1, in spite of the low species richness (Fig. 3F). Rare and dominant species were less common, and species tended to be equally abundant, during preparation and construction. At the operational stage, in contrast, two species of *Artibeus* became dominant (Fig. 4).

**Figure 5:**
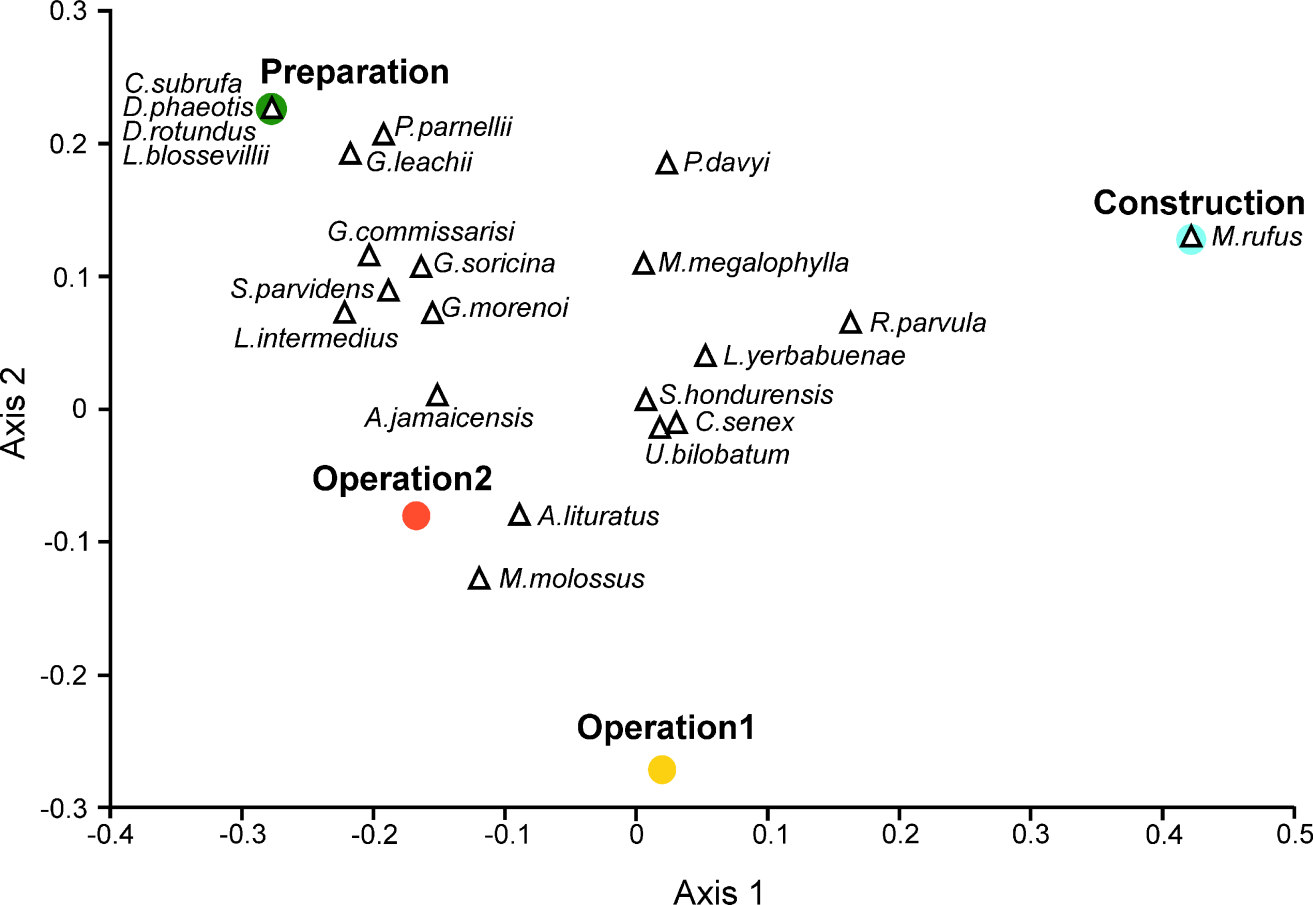
Dissimilarity of the wind farm installation stages according to their bat species composition. Principal Coordinates Analysis using the Bray–Curtis dissimilarity index for bat samples, during the site preparation, wind farm construction and two operation stages.

Species composition during construction and operational stage 1 was very different from the initial species composition, essentially due to the absence of some species (PCoA; Fig. 5). During the preparation stage we recorded four species that were absent in later monitoring: *Carollia subrufa*, *Dermanura phaeotis*, *Desmodus rotundus* and *Lasiurus blossevillii*; while *Molossus rufus* was captured only during the construction stage. One noteworthy result was that species composition during operation stage 2 was more similar to the species composition of the initial preparation stage (Fig. 5).

## Discussion

In spite of the disturbance caused by wind farm in the study area, bat species richness is relatively high in the understory (22 species). In a wind farm near our study area, Bolívar-Cimé et al. (2016) recorded 29 species of the entire bat community sampled with nets and acoustic monitoring, but of these only 14 species were recorded using mist nets. Moreover, our data reveal that, for bats flying in the understory, after the loss of diversity during construction and the first operational stage (immediate effect), bat diversity and composition by the second operational stage (delayed effect) became similar to the initial stage. As predicted, this suggests that understory bat communities are fairly resilient to the disturbances caused by wind farm installation.

Similar results have been observed in Spain, where the installation of wind farms does not affect bird or small mammal communities (De Lucas, Janss & Ferrer, 2005). Moreover, Pearce-Higgins et al. (2012) found little evidence of post-construction bird population decline in the United Kingdom, suggesting that wind farm construction can have a greater impact on birds than wind farm operation does. Also, Douglas, Bellamy & Pearce-Higgins (2011) showed that the operation of wind farms does not always negatively affect bird abundance. However, these conclusions are still not definitive given the lack of study designs that include information from before the wind farms were constructed.

In contrast, the breeding sites of seven out of nine species of grassland birds were displaced in native mixed-grass prairies during their reproductive season on wind farms in North Dakota and South Dakota, USA (Shaffer & Buhl, 2016), and wind farms may negatively affect bird abundance (De Lucas, Janss & Ferrer, 2004). Furthermore, areas with no wind turbines and those farther than 180 m from turbines had a higher density of passerine birds during operation than areas within 80 m of turbines in southwestern Minnesota (Leddy, Higgins & Naugle, 1999).

We detected immediate changes in the bat community. Six species recorded during the preparation stage were absent during construction (three insectivores, two frugivores, and one hematophague); and in the first operational stage nine of those species were absent (four insectivorous, two frugivorous, two nectarivorous and the hemotophague species). The number of captured individuals of other species also decreased, especially during construction. It was especially notorious for *P. parnellii*, which decreased from 66 captures in the preparation stage to two in the operation stage. *P. parnellii* is a species that forages near of the vegetation (Herd, 1983; Jennings et al., 2004), thus habitat loss could be the cause for the low number of captures during and after the construction stage. Jung & Kalko (2011b) classified *P. parnellii* as an urban avoider species because of the low number of detections in a forest-urban gradient in Panama. The vespertilionid *Rhogeessa parvula* increased in abundance during the construction stage, followed by reduction to the same numbers of individuals as in the preparation stage. In the region *R*. *parvula* had been found in agriculture fields by Muñoz et al. (2016), and in a mixture of pasture, thorny and palm forest by López et al. (2009); however, this species seems to be more associated to water bodies than to specific land covers (Roots & Baker, 2007). *Carollia subrufa*, *Desmodus rotundus*, *Dermanura phaeotis and Lasiurus blossevillii* were not captured during the construction or operational stages, while Molossidae species only appeared in mist nets during these stages. Bat surveys in the region (López et al., 2009; Bolívar-Cimé et al., 2016; Muñoz et al., 2016), including the present study, suggest that *Carollia subrufa* and *Dermanura phaeotis* are more sensitive to forest cover here than in other regions, because they were only recorded in primary vegetation or in the least perturbed stage. Contrarily, *Artibeus* and *Sturnira* species ranked in the first places of species captured in all stages an even increased their abundance during the two operational stages. These responses coincide with the results of Freudmann et al. (2015) and Arroyo-Rodríguez et al. (2016), who found that common and dominant frugivorous species increased in landscapes with lower forest cover. Although captures of aerial insectivorous in the understory may be due to chance, Molossidae species are frequent in cities, buildings and installations because their food—flying insects—are attracted to human facilities, including wind farms (Jung & Kalko, 2011b). There was a similar species-specific effect by turbines on bird displacement on a wind farm north-central Texas (Stevens et al., 2013). For bats in a tropical area in Mexico, phyllostomid populations also exhibited species-specific thresholds to habitat loss (Ávila Gómez et al., 2015).

One possible explanation for the decrease in functional and phylogenetic diversity during the first operational stage is that many of the bats captured belong to four large frugivorous species, and three genera had two species each (*Artibeus*, *Sturnira*, and *Glossophaga*; Table 1). The species found at this stage are considered adaptable to habitat modification because they can tolerate living in fragmented landscapes and can also benefit from recently cleared habitats (Galindo-González, 2004).

Similarly, the nine species that were present during all four stages seem to be tolerant of habitat modification (i.e., loss of vegetation cover) and to the disturbance caused by the wind farm (i.e., presence of turbines, noise, etc.). Some studies propose that adaptable species may become critical to ensure the continuity of ecological processes and the dynamics of tropical forests. For example, frugivorous bats play an important role in forest restoration in abandoned tropical pastures (Galindo-González, 2004), an ecosystem service they may be also performing in the pastures and modified habitats of our study area.

As a delayed effect, the recovery of species richness, relative abundance and composition during the second operational stage is reflected by the capture of previously recorded species: *Lasiurus intermedius* was been only caught during the site preparation stage, while *Pteronotus parnellii*, *Glossophaga commissarisi*, *G. leachii* and *Centurio senex* were not caught during the first operational stage.

These species-specific responses to landscape changes (Galindo-González, 2004) may hide community responses. For example, species richness may remain unaltered even when species composition changes, favoring generalist species in naturally (Montiel, Estrada & León, 2006) and human modified environments (Castro-Luna, Sosa & Castillo-Campos, 2007; Medina et al., 2007; Jung & Kalko, 2011a). In our study area deforestation occurred long before the wind farm installation, while during our sampling years habitat loss occurred mainly in a few large patches, as the number of patches did not increase while edge length decreased. The change of forest cover area between 2008 and 2015 represents a loss of 8% of the forest cover area, which affected mainly the areas of the vegetation patches, producing patches with less edge length. This long term deforestation may be related to the absence of species known to be sensitive to disturbance (*sensu* Galindo-González, 2004) during our monitoring (e.g., species of subfamily Phyllostominae).

In addition to the effects of forest loss, the low number of individuals captured during the construction stage may be due to the intensity and the combined effects of several factors of human disturbance, such as movement of people and machinery, noise, and the activity of turbines; all of these factors were present during both at day and night. These activities occurred only on the construction stage, so bats should have avoided the study area during this stage. Once the construction activities finished, tolerant bats such as *Artibeus* and *Sturnira* began to return. We are not neglecting the negative effects of turbines such as fatalities due to collisions, but in the understory, habitat condition and human disturbances may be more important. Greater bat species diversity has been reported for sites with more remnants of tropical deciduous and subdeciduous forest located north of a wind farm (Lira-Torres, Galindo-Leal & Briones-Salas, 2012; Briones-Salas, Peralta-Pérez & García-Luis, 2013; Briones-Salas, Cortés-Marcial & Lavariega, 2015). Taking into account that we collected 19 species with nets before the farm was built, and that there is a delayed effect leading the final species composition to be similar to that of the initial preparation stage, it is reasonable to suppose that the wind farm installation did not have a definite effect on the understory bat community. But turbine operation may have negative effects, and should still be assessed over a longer time interval.

Other studies with echolocation devices will certainly record a different community set (e.g., Bolívar-Cimé et al., 2016). Given that we focused only on the understory bats, the community structure and the conclusions derived from this work would be quite different if several height strata were sampled, using different sampling techniques (Álvarez-Castañeda & Lidicker Jr, 2015). Future studies should include the impacts of wind farms for vertical strata, to inform conservation efforts that encompass the entire bat community.

As expected, our results suggest that after the initial disturbance caused by the construction and beginning of the wind farm operation, the bat community is resilient and recovers high values of species diversity, functional diversity and phylogenetic diversity. Even though the final community sampled at the end of this study does not include exactly the same species composition of the initial stage, it could represent a steady state—albeit a less diverse one—with a potentially impoverished capacity to provide ecosystem services related to the installation of a single wind farm. But a different scenario may occur at other spatial scales. We suggest two possible conservation approaches: (1) developing specific management strategies for bat communities aimed at helping them recover a desirable stable state with higher diversity, and (2) monitoring bat communities to assess how they adapt to the new altered state. In both cases, landscape management that includes the remnants of natural vegetation, restored patches, and corridors, may be quite useful. Moreover, in the Neotropics, and particularly in the Mexican tropics, further studies of the causes of bat mortality on wind farms, population studies of species that have already been designated with a vulnerability status (e.g., *Leptonycteris yerbabuenae*), or endemic (e.g., *Glossophaga morenoi*) are all urgently needed. We also need to identify any species that may require critical attention.

##  Supplemental Information

10.7717/peerj.3424/supp-1 Supplemental Information 1 Number of bats captured over the four stages of installing a wind farm in the Mexican tropics Prep: preparation, Cons: construction, Oper1: first stage of operation (immediate effect), Oper2: second stage of operation (delayed effect). Ind: total number of individuals captured. Size (mean forearm length), Weight, Height (flight height) and Diet are functional traits whose values were obtained from literature (Bonaccorso, 1979; Ceballos & Oliva, 2005; Jung & Kalko, 2011a).

## Additional Information and Declarations

### Data Availability

The following information was supplied regarding data availability: The raw data has been supplied in Table 1.
