# Supplementary material for: Effects of a wind farm installation on the understory bat community of a highly biodiverse tropical region in Mexico
Source: PeerJ. 2017 Jun 15;5:e3424. doi: 10.7717/peerj.3424 (PMC5474091; doi:10.7717/peerj.3424)
Supplement: Supplemental Information 1 — Prep: preparation, Cons: construction, Oper1: first stage of operation (immediate effect), Oper2: second stage of operation (delayed effect). Ind: total number of individuals captured. Size (mean forearm length), Weight, Height (flight height) and Diet are functional traits whose values were obtained from literature (Bonaccorso, 1979; Ceballos & Oliva, 2005; Jung & Kalko, 2011a). [file peerj-05-3424-s001.docx]

Supporting Information

**Effects of wind farms installation on the bat community**

**of a highly biodiverse tropical region in Mexico**

Miguel Briones-Salas, Mario C. Lavariega and Claudia E. Moreno

Table S1. Resume of sampling effort spent to capture bats over four stages of a wind farm installation in the Isthmus of Tehuantepec, Oaxaca, Mexico.

| Effort | Preparation | Construction | Operation 1 | Operation 2 |
| --- | --- | --- | --- | --- |
| Number of months | 12 | 10 | 9 | 9 |
| Number of nets | 63 | 120 | 72 | 120 |
| Meters net (m) mean | 74.3 | 91.6 | 96 | 144 |
| Number of nights | 38 | 38 | 36 | 36 |
| Sampling hours ( h) | 368 | 266 | 288 | 252 |
| Total effort (net m×h) | 27,360 | 24,360 | 27,648 | 36,288 |
| Number of individuals | 425 | 89 | 154 | 251 |
| Number of species | 19 | 16 | 11 | 15 |

Table S2. Detailed sampling effort spent to capture bats over four stages of a wind farm installation in the Isthmus of Tehuantepec, Oaxaca, Mexico.

| Preparation stage | | | | | | |
| --- | --- | --- | --- | --- | --- | --- |
| Month | Year | Number of nights | Number of nets | Meters net | Sampling hours | Net m x h |
| April | 2007 | 4 | 9 | 12 | 6 | 2,592 |
| May | 2007 | 4 | 6 | 12 | 7 | 2,016 |
| Jun | 2007 | 4 | 6 | 12 | 8 | 2,304 |
| July | 2007 | 4 | 6 | 12 | 7 | 2,016 |
| August | 2007 | 4 | 6 | 12 | 8 | 2,304 |
| September | 2007 | 4 | 6 | 12 | 8 | 2,304 |
| October | 2008 | 4 | 6 | 12 | 8 | 2,304 |
| November | 2008 | 4 | 6 | 12 | 8 | 2,304 |
| December | 2008 | 4 | 6 | 12 | 8 | 2,304 |
| January | 2008 | 4 | 6 | 12 | 8 | 2,304 |
| February | 2008 | 4 | 6 | 12 | 8 | 2,304 |
| March | 2008 | 4 | 6 | 12 | 8 | 2,304 |
| Subtotal |  |  |  |  |  | 27,360 |
|  |  |  |  |  |  |  |
| Construction stage | | | | | | |
| Month | Year | Number of nights | Number of nets | Meters net | Sampling hours | Net m x h |
| November | 2010 | 4 | 6 | 12 | 7 | 2,016 |
| December | 2010 | 4 | 8 | 12 | 7 | 2,688 |
| January | 2011 | 4 | 7.5 | 12 | 7 | 2,520 |
| February | 2011 | 4 | 7 | 12 | 7 | 2,352 |
| March | 2011 | 4 | 8 | 12 | 7 | 2,688 |
| April | 2011 | 4 | 8 | 12 | 7 | 2,688 |
| May | 2011 | 3 | 8 | 12 | 7 | 2,016 |
| June | 2011 | 3 | 8 | 12 | 7 | 2,016 |
| July | 2011 | 5 | 8 | 12 | 7 | 3,360 |
| August | 2011 | 3 | 8 | 12 | 7 | 2,016 |
| Subtotal |  |  |  |  |  | 24,360 |
|  |  |  |  |  |  |  |
| Operation 1 stage | | | | | | |
| Month | Year | Number of nights | Number of nets | Meters net | Sampling hours | Net m x h |
| December | 2012 | 4 | 8 | 12 | 8 | 3,072 |
| February | 2013 | 4 | 8 | 12 | 8 | 3,072 |
| Mach | 2013 | 4 | 8 | 12 | 8 | 3,072 |
| April | 2013 | 4 | 8 | 12 | 8 | 3,072 |
| May | 2013 | 4 | 8 | 12 | 8 | 3,072 |
| June | 2013 | 4 | 8 | 12 | 8 | 3,072 |
| July | 2013 | 4 | 8 | 12 | 8 | 3,072 |
| August | 2013 | 4 | 8 | 12 | 8 | 3,072 |
| September | 2013 | 4 | 8 | 12 | 8 | 3,072 |
| Subtotal |  |  |  |  |  | 27,648 |
|  |  |  |  |  |  |  |
| Operation 2 stage | | | | | | |
| Month | Year | Number of nights | Number of nets | Meters net | Sampling hours | Net m x h |
| March | 2014 | 4 | 12 | 12 | 7 | 4,032 |
| April | 2014 | 4 | 12 | 12 | 7 | 4,032 |
| May | 2014 | 4 | 12 | 12 | 7 | 4,032 |
| June | 2014 | 4 | 12 | 12 | 7 | 4,032 |
| July | 2014 | 4 | 12 | 12 | 7 | 4,032 |
| September | 2014 | 4 | 12 | 12 | 7 | 4,032 |
| November | 2014 | 4 | 12 | 12 | 7 | 4,032 |
| December | 2014 | 4 | 12 | 12 | 7 | 4,032 |
| January | 2015 | 4 | 12 | 12 | 7 | 4,032 |
| Subtotal |  |  |  |  |  | 36,288 |
